# Determinants of breast cancer in Saudi women from Makkah region: a case-control study (breast cancer risk factors among Saudi women)

**DOI:** 10.1186/s12889-019-7942-3

**Published:** 2019-11-21

**Authors:** Fatmah J. Alsolami, Firas S. Azzeh, Khloud J. Ghafouri, Mazen M. Ghaith, Riyad A. Almaimani, Hussain A. Almasmoum, Rwaa H. Abdulal, Wesam H. Abdulaal, Abdelelah S. Jazar, Sufyan H. Tashtoush

**Affiliations:** 1 Faculty of Nursing, Umm Al-Qura University, Makkah, Kingdom of Saudi Arabia; 2 Department of Clinical Nutrition, Faculty of Applied Medical Sciences, Umm Al-Qura University, P.O. Box: 7067, Makkah, 21955 Kingdom of Saudi Arabia; 3 Department of Laboratory Medicine, Faculty of Applied Medical Sciences, Umm Al-Qura University, Makkah, Kingdom of Saudi Arabia; 4 Collage of Medicine, Department of Biochemistry, Umm Al-Qura University, Makkah, Kingdom of Saudi Arabia; 5 Department of Medical Laboratory Science, Faculty of Medical Sciences, Fakeeh College for Medical Sciences, Jeddah, Saudi Arabia; 6 Cancer Metabolism and Epigenetic Unit, Faculty of Science, King Abdulaziz University, Jeddah, Saudi Arabia; 7 Clinical Nutrition Administration, KAMC-HC, Makkah, Saudi Arabia

**Keywords:** Breast cancer, Breastfeeding practices, Economic status, Lifestyle pattern, Menstruation

## Abstract

**Background:**

There are various factors that play a major role in influencing the overall health conditions of women diagnosed with breast cancer. The population of women in Makkah region are diverse, therefore it is significant to highlight the possible determinants of breast cancer in this population. This is a case-control study that assessed determinants of breast cancer including socioeconomic factors, health-related characteristics, menstrual histories and breastfeeding among postmenopausal women in Makkah region in Saudi Arabia.

**Methods:**

A total of 432 female participants (214 cases and 218 controls) were recruited for this study. A validated questionnaire was completed by trained dietitians at King Abdullah Medical City Hospital in the Makkah region of Saudi Arabia.

**Results:**

Results displayed that determinants of breast cancer were associated significantly (*P* < 0.05) with unemployment, large family size, lack of knowledge and awareness about breast cancer, obesity, sedentary lifestyle, smoking, starting menarche at an early age, as well as hormonal and non-hormonal contraceptive use. There was no effect of diabetes, hypertension, hyperlipidemia, and duration of breastfeeding on the incidence of breast cancer.

**Conclusion:**

In summary, the results of this study accentuate the possible effect of socioeconomic factors, health-related characteristics and menstrual history on the incidence of breast cancer in postmenopausal women in the Makkah region. Education programs should be applied to increase breast cancer awareness and possibly decrease its incidence.

## Background

There has been an increasing prevalence of breast cancer among females around the world [1]. In Saudi Arabia, the recent statistics regarding women diagnosed with breast cancer are shocking. Even with the current advancements in the healthcare system and the breast cancer awareness campaign, the latest prevalence published by the Saudi Health Council in 2014 showed that breast cancer accounted for 29% of all the cancer types diagnosed in women. Unfortunately, few women present with early stages of the disease, compared to a substantial proportion of women who present in the late stages of breast cancer, when the tumour has become metastatic [2].

Previous studies have reported that there are several common factors present in women diagnosed with breast cancer, such as their ages, ages at menarche and menopause, family histories, lifestyles and oral contraceptive usage [3, 4]. However, the presentation of these factors varies among different populations of women. A greater number of breast cancer diagnoses have been linked to variances in the lifestyle patterns and socioeconomic factors. From the point of view of epidemiological studies, exploring the predominant risk factors in a selected population of women can help to direct the perspective of breast cancer prevention [4].

The population of women in the region of Makkah is diverse, with different lifestyle patterns, economic statuses and breastfeeding practices. These factors play significant roles in influencing the overall health conditions and make it an area of interest for investigating the determinants of breast cancer in this specific population. Furthermore, postmenopausal women were more likely to have breast cancer than premenopausal women [5]. Therefore, the aim of this study was to explore which of the socioeconomic factors, health-related characteristics, menstruation starting and ending ages and breastfeeding histories were determining factors for postmenopausal women diagnosed with breast cancer.

## Methods

### Study design and setting

This case-control study was conducted from June 2014 through November 2016 at King Abdullah Medical City Hospital (KAMC) in the Makkah region of Saudi Arabia. This hospital is the only centre that provides cancer screening and treatment for residents in Makkah region.

### Participants

A total of 432 female participants (214 cases and 218 controls) were recruited for this study. We included postmenopausal Saudi women of Arabic ethnicity aged > 45 years from the Makkah region who were newly diagnosed with breast cancer that was biopsy confirmed by a cancer pathologist in KAMC. Studies have showed that factors associated to breast cancer differs in racial groups [6, 7]. Therefore, we excluded women of any other nationality and African-Asians ethnicity. We also not included any breast cancer women diagnosed with any other type of cancer and who had a metastatic (stage IV) and/or recurrent breast cancer. Any woman stopped her menstrual periods within the last 12 months was defined as postmenopausal. The women in the control group were made up of hospital workers and the patients’ companions and friends. The controls were selected from the same region of cases and matched on a single year of age for both groups. Based on the above exclusion and inclusion criteria, 214 out of 229 cases were included in this study. However, some patients and healthy individuals were not recruited in this study due to; non-Saudi nationality (*n* = 7 cases and 12 controls), African-Asians ethnicity (*n* = 3 cases and 4 controls), premenopausal women and/or aged < 45 years (*n* = 2 cases), metastatic breast cancer diagnosis (*n* = 1 case), recurrent breast cancer (*n* = 1 case), and diagnosis of multiple cancer types (*n* = 1 case).

### Data collection

Convenience sampling was used to collect the data for this study. As a routine work in the hospital, all newly diagnosed cancer patients should meet a registered dietitian to evaluate his/her nutritional status. During this evaluation, a self-administered questionnaire was completed by each of the participants via a face-to-face interview. The socioeconomic factors, health-related characteristics, menstrual histories as well as breastfeeding duration tested in this study were part of a previously validated questionnaire developed by Wilson et al. (2013) [8] that focused on well-known determinants associated with breast cancer in postmenopausal women. Each participant’s body mass index (BMI; kg/m^2^) was calculated after measuring the weight and height in the hospital and at the time of the data collection. Any participant with a BMI < 18.5 kg/m^2^ was classified as underweight, normal weight was 18.5–24.9 kg/m^2^, overweight was 25–29.9 kg/m^2^ and obese was > 30 kg/m^2^.

### Statistical analysis

All of the statistical tests were completed using IBM SPSS Statistics for Windows version 20.0 (IBM Corp., Armonk, NY, USA), and a *P*-value < 0.05 was set for the significant differences. The Kolmogorov-Smirnov normality test was used to determine the normality of distribution. The *P*-value for each parameter was determined using a suitable test, which is mentioned as a footnote in each table. In order to ascertain the differences between the cases and the controls, the data from the participants was stratified using a case-control status. A chi squared test and t-test were conducted for the parametric and nonparametric variables to determine the differences in the socioeconomic factors, health-related characteristics, menstrual histories and breastfeeding durations.

To determine the possible risk factors related to breast cancer, the odds ratio (OR), 95% confidence interval (95% CI) and β-coefficient were determined by using a logistic regression test. All of the variables were adjusted for potential confounders; age (continuous), BMI (continuous), employment, family income, education, family size, marital status, physical activity, smoking, family history of breast cancer, other health problems, contraceptive use, age at menarche, age at menopause, and breastfeeding duration.

## Results

An overview of the socioeconomic characteristics of the participants is presented in Table 1. The participants’ ages ranged from 45 to 75 years old, and the mean ages for the case and control groups were 57 ± 7.3 years old and 56.9 ± 8.6 years old, respectively. The results showed significant differences regarding some of the socioeconomic factors (*P* < 0.001), such as employment, income, education and family size.

**Table 1:** Socioeconomic characteristics of the study groups

Parameter	Control	Case	*P*-value
Number [n (%)]	218 (50.5%)	214 (49.5%)	0.847
Age (year)	56.9 ± 8.6	57 ± 7.3	0.526
Employment			
Yes	178 (81.7%)	56 (26.2%)	< 0.001
No	40 (18.3%)	158 (73.8%)	
Family income			
< 5000 SR^a^	21 (9.6%)	94 (43.9%)	< 0.001
5000–10000 SR	85 (39%)	88 (41.1%)	
10000–20000 SR	80 (36.7%)	24 (11.2%)	
> 20000 SR	32 (14.7%)	8 (3.8%)	
Education			
Illiterate	2 (0.9%)	32 (15%)	< 0.001
Primary	3 (1.4%)	96 (44.9%)	
Intermediate/secondary	23 (10.6%)	38 (17.8%)	
Postsecondary	190 (87.1%)	48 (22.3%)	
Family size			
5 or less	114 (52.3%)	40 (18.7%)	< 0.001
6 or more	104 (47.7%)	174 (81.3%)	
Marital Status (Married)			
Yes	191 (87.6%)	204 (94.1%)	0.087
No	27 (12.4%)	10 (5.9%)	

Values are expressed as frequency (%) or Mean ± SD

*P*-values are obtained by t-test for the parametric variable (age) or *x*^2^ for non-parametric variables

^a^*SR* Saudi Riyal

The highest employment status percentage in both groups was 81.7% employed participants in the control group, with 73.8% unemployed in the case group. Nearly one-half of the participants in the case group (43.9%) fell in the low-income category of < 5000 Saudi Riyal (SR) of monthly income (~ 1333.17 American Dollar) when compared to the control group (9.6%). Both groups had low percentages in the highest income category of > 20,000 SR (~ 5332.70 USD): 14.7% for the control group and 3.8% for the case group.

The illiteracy rate was higher among the cases (15%) when compared to the control group (0.9%). All of the participants in both groups reported varied results in obtaining an education, with a higher result for postsecondary education of 87.1% for the control group, compared to 22.3% for the case group for the same level of education. Having a large family size (6 or more family members) was more common in the case group (81.3%), while the control group showed no noticeable difference in the percentages of having small or large family sizes (52.3 and 47.7%, respectively). There were no significant differences in the marital statuses in either group (*P* > 0.05); the percentages of married participants were fairly high in both groups (87.6% for the controls and 94.1% for the cases).

With regard to the health-related characteristics for the participants in this study (Table 2), the BMI (*P* < 0.001), regular exercise (*P* = 0.009), cancer awareness (*P* < 0.001), smoking (*P* < 0.001), diabetes (*P* < 0.001), hypertension (*P* < 0.001) and the use of contraceptives (*P* < 0.001) were significant when testing the differences between the groups. Based on these results, it was clear that there was a higher BMI (obese category) percentage of 63.6% among the cases when compared to the control group (24.3%). It was also evident that regular exercise was practiced among few of the participants in both the control and case groups (37.2% vs. 26.2%, respectively). Overall, high percentages of the participants were aware of cancer (98.2% for the control group and 81.3% for the case group). Although the results of having a family history of breast cancer were not significant (*P* > 0.05), the family history results of the patients with breast cancer were higher in the cases (17.8%) than in the participants in the control group (6%). The smoking status showed that 17.8% of the participants in the case group were smokers, compared to 1.4% being smokers in the control group.

**Table 2:** Health-related characteristics of the study groups

Parameter	Control (*n* = 218)	Case (*n* = 214)	*P*-value
Weight (kg)	69.5 ± 14.7	88.5 ± 17.5	< 0.001
Height (cm)	158.6 ± 7	157.7 ± 6.7	0.637
Body Mass Index (BMI) (kg/m^2^)	27.7 ± 6.3	35.4 ± 10	< 0.001
BMI categories			
Underweight	1 (0.4%)	0	< 0.001
Normal	69 (31.7%)	22 (10.2%)	
Overweight	95 (43.6%)	56 (26.2%)	
Obese	53 (24.3%)	136 (63.6%)	
Cancer awareness			
Yes	214 (98.2%)	174 (81.3%)	< 0.001
No	4 (1.8%)	40 (18.7%)	
Regularly exercise			
Yes	81 (37.2%)	56 (26.2%)	0.009
No	137 (62.8%)	158 (73.8%)	
Family history of breast cancer			
Yes	13 (6%)	38 (17.8%)	0.072
No	205 (94%)	176 (82.2%)	
Smoking			
Yes	3 (1.4%)	38 (17.8%)	< 0.001
No	215 (98.6%)	176 (82.2%)	
Diabetes			
Yes	17 (7.8%)	72 (33.6%)	< 0.001
No	201 (92.2%)	142 (66.4%)	
Hypertension			
Yes	33 (15.1%)	104 (48.6%)	< 0.001
No	185 (84.9%)	110 (51.4%)	
Hyperlipidemia			
Yes	28 (12.8%)	40 (18.7%)	0.062
No	190 (87.2%)	174 (81.3%)	
Contraceptive use			
Hormonal	55 (25.2%)	94 (43.9%)	< 0.001
Not-hormonal	31 (14.2%)	32 (15%)	
Don’t use	132 (60.6%)	88 (41.1%)	

Values are expressed as frequency (%)

*P*-values are obtained by Mann-Whitney test for non-parametric continuous variables (weight, height and BMI) or by x2 test for discontinuous variables

Additionally, the percentage of breast cancer patients diagnosed with diabetes was higher (33.6%) than the diabetic participants in the control group (7.8%). Similarly, hypertension was higher in the cases when compared to the control group (48.6% vs. 15.1%, respectively). The screening for positive hyperlipidaemia results showed no significant difference between the two groups, but the percentage was low in the participants in the control group (12.8%) when compared to the cases (18.7%). The use of hormonal contraceptive types was higher in the cases (43.9%), whereas the highest percentage in the control group (60.6%) included those participants not using any contraceptive methods.

The results of the menstruation histories and breastfeeding durations are shown in Table 3. Both groups reported higher menstruation percentages at the ages of 11–14 years old; 70.1% of the cases and 89.9% of the control group began menstruation around this age. High percentages in both groups exhibited breastfeeding histories, with the results showing that most of the cases (70%) breastfed for a duration of 6–12 months, while most of the participants in the control group breastfed for a duration of less than 6 months. The results of the age of menopause showed no statistical difference between the groups. The highest percentage was 46 years old and older for 67.3% of the cases and 56.4% of the controls.

**Table 3:** Menstrual history and breastfeeding duration of the study groups

Parameter	Control (*n* = 218)	Case (*n* = 214)	*P*-value
Age of started menstruation			
< 10 years old	1 (0.5)	25 (11.7%)	< 0.001
11–14 years old	196 (89.9%)	150 (70.1%)	
> 15 years old	21 (9.6%)	39 (18.2%)	
Age at menopause			
< 35 years old	2 (0.9%)	8 (3.7%)	0.271
36–40 years old	25 (11.5%)	16 (7.5%)	
41–45 years old	68 (31.2%)	46 (21.5%)	
> 46 years old	123 (56.4%)	144 (67.3%)	
Breastfeeding duration			
No	51 (23.4%)	40 (18.7%)	0.052
Yes	167 (76.6%)	174 (81.3%)	
< 6 months	76 (45.5%)	48 (27.6%)	
6–12 months	42 (25.1%)	70 (40.2%)	
> 13 months	49 (29.4%)	56 (32.2%)	

Values are expressed as frequency (%)

Duration of breast feeding is average for each pregnancy

*P*-values are obtained by *x*^2^ test

The correlations between the potential dependent variables for breast cancer are shown in Table 4. With regard to the socioeconomic factors, the results showed that being unemployed had an increased positive association with breast cancer (β = 1.89, OR = 6.56, 95% CI = 3.83–11.37, *P* < 0.001). This was similar to the results of being in the low-income category of < 5000 SR (~ 1333.17 USD) (β = 3.69, OR = 39.88, 95% CI = 11.11–143.16, *P* < 0.001). Additionally, the results showed a positive association between having a large family size (6 members and more) and breast cancer (β = 0.8, OR = 2.23, 95% CI = 1.15–4.3, *P* = 0.017). Moreover, having a primary level of education had a positive association with breast cancer (β = 4.07, OR = 58.56, 95% CI = 16.9–202.82, *P* < 0.001).

**Table 4:** Potential significant predictors related to breast cancer

Independent variable	β	OR	95% CI	*P*-value
Body Mass Index (BMI) (continuous)	0.1	1.11	1.07–1.14	< 0.001
BMI categories				
Underweight	ND	ND	ND	ND
Normal	0	1		
Overweight	0.41	1.5	0.77–2.93	0.234
Obese	1.39	4	2.07–7.74	< 0.001
Employment				
Yes	0	1		
No	1.89	6.56	3.83–11.37	< 0.001
Family income				
< 5000 SR^a^	3.69	39.88	11.11–143.16	< 0.001
5000–10000 SR	2.07	7.88	2.45–25.4	0.001
10000–20000 SR	1.36	3.89	1.09–13.88	0.036
> 20000 SR	0	1		
Education				
Illiterate	2.98	19.7	4.33–89.54	< 0.001
Primary	4.07	58.56	16.91–202.82	< 0.001
Intermediate-secondary	1.71	5.52	2.75–11.09	< 0.001
Postsecondary	0	1		
Family size				
5 or less	0	1		
6 or more	0.8	2.23	1.15–4.3	0.017
Regularly exercise				
Yes	0	1		
No	0.72	2.06	1.16–3.67	0.014
Cancer awareness				
Yes	0	1		
No	1.87	6.47	1.84–22.77	0.004
Smoking				
Yes	1.85	6.36	1.56–26	0.01
No	0	1		
Diabetes				
Yes	0.5	1.65	0.81–3.35	0.165
No	0	1		
Hypertension				
Yes	0.51	1.67	0.91–3.04	0.097
No	0	1		
Contraceptive use				
Hormonal	1.91	6.78	3.42–13.44	< 0.001
None-hormonal	1.23	3.43	1.54–7.65	0.003
Don’t use	0	1		
Age of started menstruation				
< 10 years old	1.61	5	1.12–22.29	0.035
11–14 years old	0	1		
> 15 years old	0.54	1.57	0.79–3.13	0.197

All variables were adjusted for potential confounders

*Abbreviations*; *β* Beta coefficient, *CI* Confidence Interval, *ND* Not Determined, *OR* Odds Ratio

^a^SR Saudi Riyal

The health-related characteristics, such as the BMI, exhibited positive correlations to breast cancer (β = 0.1, OR = 1.11, 95% CI = 1.07–1.14, *P* < 0.001), which was significant in the obese BMI category (β = 1.39, OR = 4, 95% CI = 2.07–7.74, *P* < 0.001). Additionally, having an awareness about breast cancer and the smoking status were significant factors correlated with breast cancer. Having no awareness was positively associated with breast cancer (β = 1.87, OR = 6.47, 95% CI = 1.84–22.77, *P* = 0.004), while being a smoker showed an increased risk of breast factor in this study (β = 1.85, OR = 6.36, 95% CI = 1.56–26, *P* = 0.01). Moreover, the use of both types of contraceptive methods, hormonal and nonhormonal, increased the risk of breast cancer. In fact, the higher risk was involved in using a hormonal type of contraception (β = 1.91, OR = 6.78, 95% CI = 3.42–13.44, *P* < 0.001).

Beginning one’s menstruation cycle at an early age (10 years old or less) increased the risk of breast cancer, according to the results of this study (β = 1.61, OR = 5, 95% CI = 1.12–22.29, *P* = 0.035). The not significantly identified variables by regression test; overweight, diabetes, hypertension and age of stated menstruation > 15 years old, were not included in this study as determinants of breast cancer.

## Discussion

The current study investigated the effects of socioeconomic factors, health related characteristics, menstrual histories and breastfeeding durations on the incidence of breast cancer in postmenopausal Saudi Arabian women from the Makkah region.

### Low-income, family size and employment

The current study showed significant findings regarding the association between being unemployed and having breast cancer, which were similar to those of having a large family and a low income. Being unemployed itself is a risk factor for having a low income, which is one of the major obstacles in the early detection of breast cancer. In addition, the cost of diagnostic procedures is a challenge in breast cancer prevention [9]. In the current study, the majority of the women in the cases group were unemployed, which can be a clear reason for some of them being in the low-income category. In addition, an increased family size can increase the responsibility and overwhelm a family financially, which in turn, can be one reason for not being able to afford the cost of engaging in early breast cancer detection programs. Those cases with an increased family size in the current study were already diagnosed with breast cancer and receiving treatment, reflecting the high possibility of not having had previous preventive screenings. Unfortunately, with the increasing prevalence of breast cancer, there are still few healthcare services in Saudi Arabia that provide free screening, creating further obstacles for low-income women [10]. Trieu et al., [11] reported that postmenopausal women with low family size were more likely to have breast cancer than women with large family size, and this result was not in line with the study results. These conflicting results could be related to the increase ratio of breastfeeding with higher number of babies; consequently, decrease the possibility of breast cancer incidence. However, some mothers in Makkah do not like to breastfeed their babies and alternatively introduce complementary foods and/or bottle-feeding at an early age of baby’s life [12], which could increase the probability of breast cancer incidence in postmenopausal women [13]. These erroneous practices with large family size could be a risk factor for breast cancer.

### Education and Cancer awareness

The lack of knowledge and awareness about breast cancer was a confirmed risk for the increasing prevalence of breast cancer. Being unaware of breast cancer showed a positive relationship toward an increased breast cancer risk in the current study (Table 4). The level of awareness of the cases in this study was high, which is a promising value. This high number may be because breast cancer campaigns are conducted every year in Saudi Arabia on and around the breast cancer awareness day in order to spread awareness [2, 14]. Therefore, information regarding breast cancer is widely available for everyone to access via different media; however, information about breast cancer can be of less use to illiterate women. Although the percentage of women with breast cancer who had no formal education was small when compared to the total number in the group, it still highlighted the need to consider tailored interventions for this group [15]. Health literacy remains a social determinant of health that affects both educated and uneducated women [16].

### Obesity and exercise

Research has provided evidence that a sedentary lifestyle can affect many aspects of health, such as the risk of obesity [17]. The results of this study showed that the rate of obesity was higher in the newly diagnosed cases with breast cancer, and that being in the higher BMI category increased the incidence of breast cancer 4-fold when compared to the normal weight women. These results are in line with other findings that identified a positive association between obesity and breast cancer [18, 19]. There are two reasons for these findings in newly diagnosed patients. The first reflects the nature of the fat tissues (adipose) in obese individuals, which produce inflammatory cytokines and certain chemical mediators that assist in prompting cancer cell invasion and metastasis [20]. The second reason could be related to the gradual increase in weight while a patient is receiving cancer treatment. In newly diagnosed patients with breast cancer, a weight gain ranging from 1.0 kg to 6.0 kg has been identified in the first year after establishing treatment [21]. However, the risks contributed to obesity have become major public health problems in Saudi Arabian women [22], with more attention being paid to the trend of increasing obesity with increasing age [23].

Exercise or physical activity could be implemented to overcome the high obesity prevalence. Exercise means a scheduled training to achieve a specific purpose, while physical activity means movement of skeletal muscle that requires some energy and can include routine daily activities [24]. A lack of physical activity is a complicated facet of living a sedentary lifestyle, and the current study showed that the majority of the breast cancer cases and controls were not engaged in regular exercise routines. Regular exercise has been shown to be a significant factor that is positively associated with a decreasing incidence of breast cancer in several different studies [24, 25], similar to this study (Table 4). During the distinct stages of breast cancer, whether preventive, during treatment or post-treatment recovery, engaging in physical activity has shown its positive impact in mediating tumorigenesis and its effects on the body [26]. A sedentary lifestyle is an alarming risk on Saudi Arabian women’s health conditions, and interventions promoting physical activities are essential [27, 28].

### Smoking

The risk of smoking on the development of tumours has been tested in several types of cancer, and most of the published results have shown a positive association [29]. Being a smoker in this study showed a positive association with an increased risk of breast cancer of about 6 times that of nonsmokers. Smoking has been linked to an underlying tumour progression mechanism, and increased epithelial-to-mesenchymal transition and motility have been observed in breast tumour cells after they have been exposed to cigarette smoke [29]. One cohort study that investigated the association between smoking and developing breast cancer found that among the 102,927 women recruited for the study, during the 788,361 person-years (mean = 7.7 years) of follow up, 1815 women developed invasive breast cancer [30]. Their findings also showed that the significance of breast cancer increased when the women started smoking at an early age (< 17-year-old) [30].

### Menarche

The reproductive age for women is a time when many hormonal changes arise in the body; therefore, direct effects on the function and development of breast tissue can occur. The rapid increase in the production of steroid hormones when starting menstruation (menarche) is highly associated with an increased risk of breast cancer [31]. One meta-analysis study including 118,964 women with breast cancer (cases) and 306,091 without the disease reported that the younger the age at which a woman began menstruation contributed to a greater risk of developing breast cancer, with a risk ratio of 1.05 for every year younger at the time of menarche [32]. This study revealed that starting menarche at an early age (10 years or less) increased the risk for breast cancer by 5 times when compared to those women who started at a normal age [32]. Since the beginning of the reproductive age varies among women, breast cancer preventive measures will have a greater effect if they are initiated early in a woman’s life [32].

### Contraceptives

Obviously, the effects of the hormonal changes on a woman’s body during reproductive age can increase the risk of breast cancer, and the effects of synthetic hormones are no exception. Several studies have found a reverse effect of contraceptives on breast tissue [33–35]. The combined estrogen in hormonal contraception has been identified in earlier studies as a major cause for breast cancer [33, 34]. One cohort study that followed 1.8 million women for an average of 10.9 years showed that 11,517 cases of breast cancer occurred in those women using hormonal contraception when compared to those who did not use any contraceptive methods [34]. Those study results showed a relative risk of 120 for the hormonal contraceptive users when compared to the nonusers (95% CI = 1.14–1.26) [34]. The current study agrees with the previous findings by showing an increased risk of 6.78 times for the hormonal contraceptive users when compared to the nonusers. Knowing the association between the hormonal contraceptive usage time and the diagnosis of breast cancer in this study can reveal other findings; however, using hormonal contraception for less than 1 year remains a risk factor for developing breast cancer [34].

This study was limited by the convenience sampling technique, the regional sample collection, and postmenopausal women recruitment. Other determinants of breast cancer are recommended to study in Makkah region, such as dietary habits and breast density. However, it does highlight the importance of determining the risk factors for breast cancer using cohort studies consisting of a nationwide and large sample size, such studies are limited. Therefore, due to the paucity of research concerning of breast cancer in Makkah region, this study adds to the body of knowledge for future research to expand on this area.

## Conclusion

This study showed that most of the socioeconomic, health related status and menstruation history variables were determinant risk factors for breast cancer in postmenopausal women in the Makkah region. To illustrate, education, economic status, obesity, lack of exercise, cancer awareness, smoking, hormonal and non-hormonal contraceptive use and an early menstruation age were identified as significant in this study, and they should be considered in culturally sensible prevention programs for women in the Makkah region of Saudi Arabia.

## Data Availability

The datasets used and/or analyzed in this research cannot be publicly shared and they are available from the corresponding author on reasonable request.

## Abbreviations

BMI: Body mass index
CI: Confidence interval
CRC: Colorectal cancer
KAMC: King Abdullah Medical City Hospital
OR: Odds ratio
*x*^2^: Chi-squared
